# Severe Sepsis Associated With Multiorgan Failure and Precipitating Nonhepatic Hyperammonemia Crisis in Late-Onset Ornithine Transcarbamylase Deficiency: A Case Report and Literature Review

**DOI:** 10.7759/cureus.55711

**Published:** 2024-03-07

**Authors:** Sabastain F Forsah, Derek Ugwendum, Divine Besong Arrey Agbor, Nkafu Bechem Ndemazie, Gauvain Kankeu Tonpouwo, Nancelle Ndema, Akua Aboah Taylor, Jay Nfonoyim

**Affiliations:** 1 Internal Medicine, Richmond University Medical Center, Staten Island, USA; 2 Internal Medicine, JC (Jean-Charles) Medical Center, Orlando, USA; 3 Pulmonary and Critical Care, Richmond University Medical Center, Staten Island, USA

**Keywords:** multiorgan failure, hyperammonemia, ornithine transcarbamylase deficiency, septic shock, sepsis

## Abstract

Sepsis is characterized by a dysregulated immune response to an infection. It is a major public health problem owing to its high mortality and morbidity. Sepsis is a medical emergency and requires aggressive and timely management. It can cause multiorgan failure, unmask an existing but undiagnosed disease such as ornithine transcarbamylase deficiency (OTCD), or make a known well-controlled disease worse. We present the case of a 52-year-old male who was brought to the emergency department unresponsive. He was diagnosed with severe sepsis which was associated with multiorgan failure and hyperammonemia crisis. Hyperammonemia was due to a newly diagnosed, late-onset OTCD which was unmasked by severe sepsis. This case will enable physicians to be aware and consider OTCD in a patient presenting with severe sepsis, altered mentation, and seizures, with no obvious cause of hyperammonemia.

## Introduction

Globally, sepsis accounts for about 20% of deaths, with most cases requiring intensive care unit (ICU) admission [1]. It is a major public health problem owing to its high morbidity and mortality [2]. Sepsis is a medical emergency with guidelines recommending aggressive and timely management because a patient can rapidly deteriorate into multiorgan failure [3]. Sepsis can also unmask an existing, but undiagnosed disease. One such condition is ornithine transcarbamylase deficiency (OTCD) which can cause significantly elevated serum ammonia levels, with the risk of developing cerebral edema and brain herniation [4]. Symptoms of OTCD usually develop after a stressful event like an acute infection [5]. In this case presentation, we have a male patient with no knowledge about his OTCD, who presented with severe sepsis and as a result developed multiorgan failure and severe symptomatic hyperammonemia without significant liver dysfunction. Further testing revealed that he had OTCD. This is a unique case of severe sepsis progressing to septic shock with multiorgan failure and precipitating a hyperammonemic crisis in a patient with an undiagnosed OTCD.

## Case presentation

A 52-year-old male with a past medical history of stage 3 chronic kidney disease, type 2 diabetes mellitus, hepatitis B infection, and hypertension and with no known history of seizures was brought to the emergency department unresponsive. According to his wife, the patient had no signs and symptoms the days prior. He had two generalized, tonic-clonic seizures en route to the hospital and one more seizure in the emergency room with the latter lasting for about four minutes. The patient’s primary care doctor reported that six months before his current presentation, he had no complaints and his blood work was grossly normal. 

Initial vital signs showed a blood pressure of 109/70 mmHg; heart rate of 131 beats per minute; respiratory rate of 26 breaths per minute; and temperature of 96.1oF. He was cyanotic and unresponsive to noxious stimuli, with pupils sluggishly reactive to light. Corneal and gag reflexes were present. His abdomen was distended and soft with hypoactive bowel sounds. The rest of his physical examination was unremarkable. The quick Sequential Organ Failure Assessment (qSOFA) score was 2, due to the patient's respiratory rate being >22 breaths per minute and his altered mental status.

Initial key laboratory findings are shown in Table 1. It shows significantly elevated ammonia, creatine kinase, and lactic acid levels with a very high anion gap metabolic acidosis. He also had leukocytosis while the liver enzymes were within normal limits.

**Table 1:** Initial laboratory findings Alk phos, alkaline phosphatase; ALT, alanine aminotransferase; AST, aspartate aminotransferase; BUN, blood urea nitrogen; CO_2_, carbon dioxide; HB, hemoglobin; pO2, T. bili, total bilirubin; WBC, white blood count.

Parameters	Patient’s results	Reference value
Hematology		
WBC (K/UL)	14,2	4-11.2
Hb (g/dl)	16.3	13.7-17.5
Platelet (k/UL)	183	150-400
Biochemistry		
Sodium (mmol/L)	148	136-145
Potassium (mmol/L)	3.9	3.5-5.1
Chloride (mmol/L)	98	98-108
CO_2_ (mmol/L)	<10	20-31
Anion gap (mmol/L)	45	10-20
BUN (mg/dl)	23	7-18
Creatinine (mg/dl)	2.3	0.7-1.3
T bili (mg/dl)	0.6	0.2-1
AST (U/L)	29	<34
ALT (U/L)	42	10-49
Lactic acid (mmol/L)	29	0.5-2.2
Ammonia (umol/L)	870	11-32
High-sensitivity troponin (ng/L)	96.6	< 55
Creatine kinase (U/L)	102460	46-171
Coagulation factors		
PT(sec)	13	12-14.8
PTT(sec)	33	22.8-36.5
INR	1.1	0.9-1.12

Additionally, the urine drug screen was negative; serum alcohol, acetaminophen, and salicylate levels were undetectable, and there were no features suggestive of a urinary tract infection.

**Figure 1:**
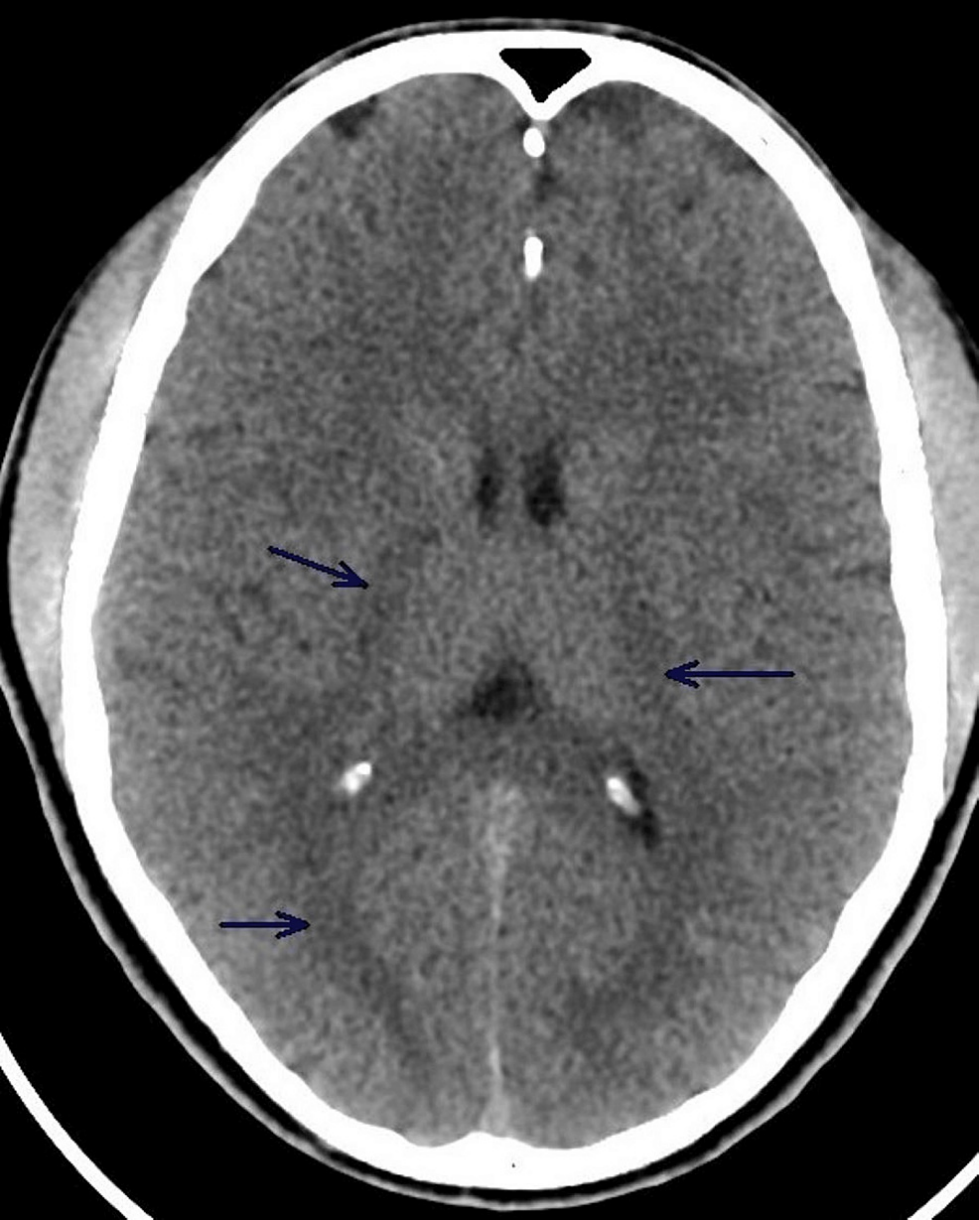
CT of the brain showing no acute abnormalities

His electrocardiogram (EKG) showed normal sinus rhythm and bedside echocardiogram showed normal left ventricular function with no wall motion or valvular abnormalities. The computed tomography (CT) scan of the brain showed no acute abnormalities (Figure 1).

**Figure 2:**
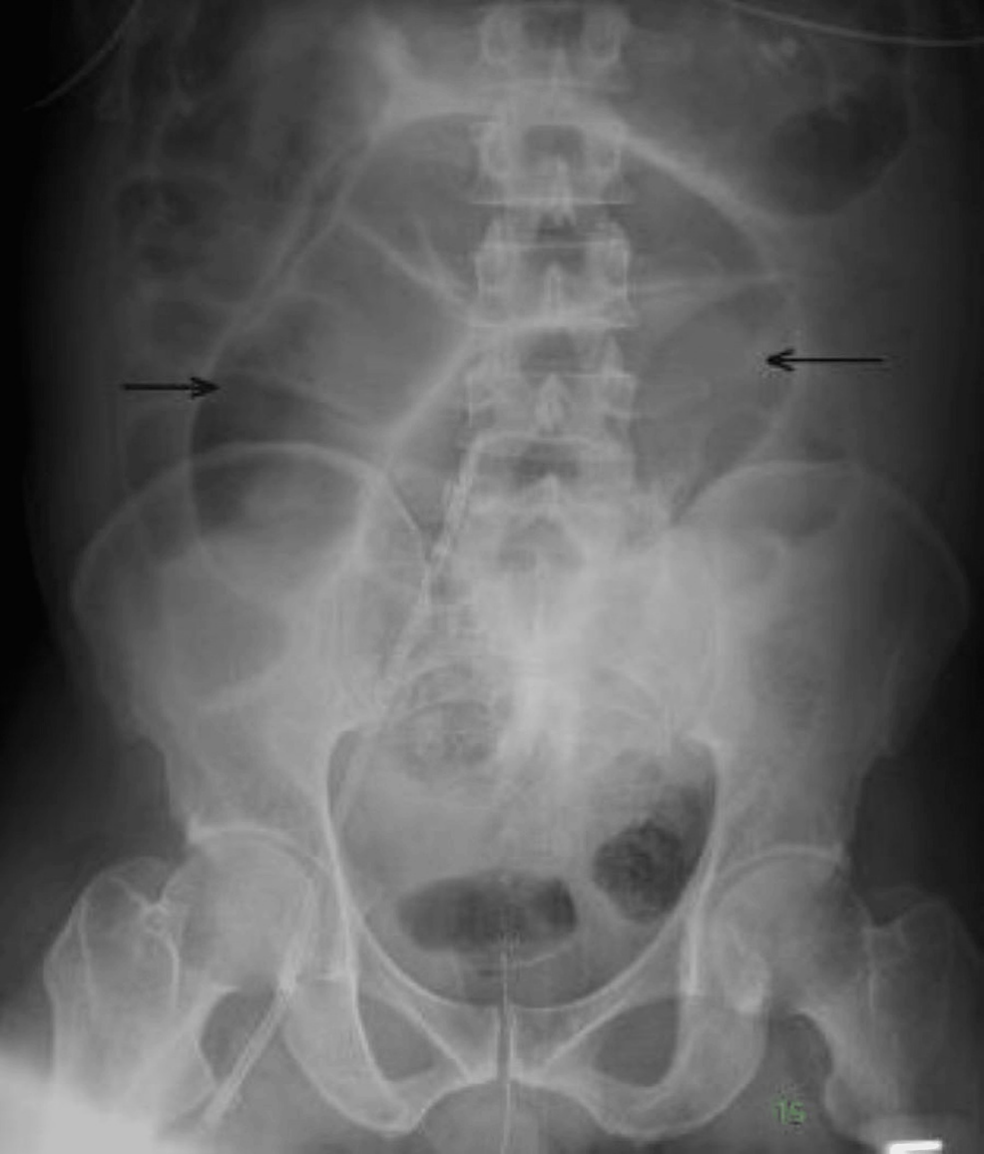
Abdominal X-ray showing the dilated colon

Abdominal X-ray showed moderate fecal stasis and dilation of the colon with no intestinal obstruction and no intraperitoneal free air (Figure 2).

**Figure 3:**
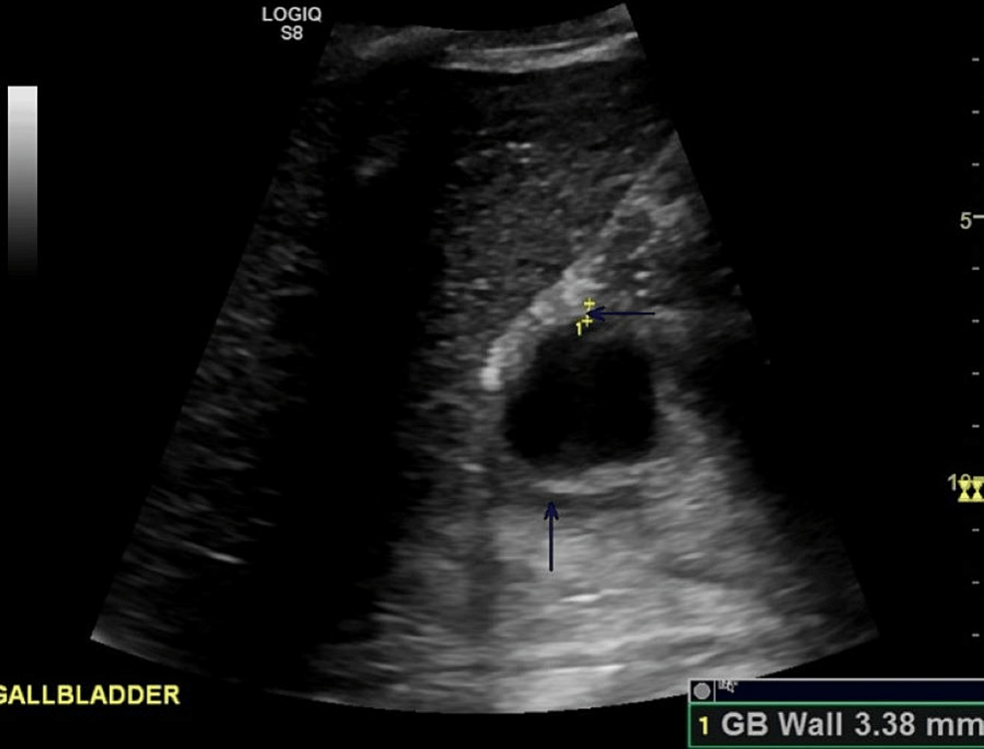
Abdominal ultrasound showing gallbladder wall thickening and pericholecystic fluid

Abdominal ultrasound showed biliary sludge, gallbladder wall thickening, and pericholecystic fluid, suggesting cholecystitis. The liver was normal in size, echotexture, and without a focal hepatic lesion (Figure 3).

The patient was intubated for airway protection, mechanically ventilated, sedated with propofol, and admitted to the ICU. He became hypotensive but after fluid resuscitation, his blood pressure did not improve, requiring norepinephrine to maintain a mean arterial pressure above 65mmHg. Lactulose was administered through an orogastric tube (OGT) to enhance bowel movement. A sodium bicarbonate drip was initiated for severe metabolic acidosis and broad-spectrum antibiotics were administered after blood cultures were obtained. Plasma amino acid levels, urine organic acid, and urea cycle enzyme testing were done because of the significantly high ammonia levels. Electroencephalography (EEG) showed diffuse slowing with no epileptiform discharges.

Urgent hemodialysis (HD) was done due to severe hyperammonemia and a repeat ammonia level after HD was 207umol/L. His kidney function, liver enzymes, electrolytes, troponin, lactic acid, creatine kinase, and ammonia levels were monitored every 12 hours and post HD as shown in Table 2.

**Table 2:** Trends of selected laboratory values during the patient’s course of admission ALT, alanine aminotransferase; AST, aspartate aminotransferase; Cr, creatinine; Hb, hemoglobin; T.bili, total bilirubin; INR, internal normalized ratio; HD, hemodialysis

Time	Reference values	Initial labs	After first HD	12 hours after admission	24 hours after admission	36 hours after admission
Ammonia (umol/L)	11-32	870	207	507	723	353
Lactic acid (nmol/L)	0.5-2.2	29	20.3	19.4	21.1	24.7
Creatine kinase (U/L)	0.7-1.3	102400	16,045	204494	151882	14583
ALT (U/L)	10-49	42	535	1196	8612	13615
AST (U/L)	<34	29	575	2041	18589	24032
T. Bili (mg/dL)	0.2-1	0.6	0.5	1.3	2.1	3.4
INR	0.9 – 1.12	1.1	8.3	>20	10.52	8.04
PTT (sec)	22.8-36.5	13	86	>200	171	143
High sensitivity troponins (ng/L)	<55	96.6	3787	3087	2235	1923
Hb (g/dl)	13.7-17.5	16.3	14.1	12.4	7.4	8.5
Platelets	150-400	183	97	57	37	41
Cr (mg/dL)	0.7-1.3	2.3	2.87	3.41	3.47	4.61
Potassium (mmol/L)	3.5- 5.1	3.9	6.1	5.3	6.3	5.1

Due to worsening abdominal distention, his OGT was connected to intermittent suction, and approximately 1300mls of bloody gastrointestinal fluid was aspirated. Fibrinogen level was <60mg/dl, prompting the administration of prothrombin complex concentrate and the transfusion of pack red blood cells and platelets due to the high risk of severe bleeding with elevated international normalized ratio (INR), acute blood loss anemia, and severe thrombocytopenia, respectively.

His short hospital course was marked by anuria, high oxygen requirement, dissemination intravascular coagulation, and loss of brain stem function. About 38 hours after admission, the patient went into a cardiopulmonary arrest, and resuscitation attempts were unsuccessful.

Delayed results showed negative blood cultures; the patient however had OTCD, which was a new diagnosis in this patient.

## Discussion

Globally, about 49 million patients were diagnosed with sepsis resulting in 11 million deaths in 2017 [1]. Sepsis is an acute life-threatening, dysregulated immune response to an infection leading to physiological, pathological, and biological abnormalities. Patients with sepsis, if left untreated, may progress to severe sepsis which is characterized by organ dysfunction involving either a single organ or multiple organs. As part of the pathophysiology of sepsis, some patients deteriorate further to septic shock, in which case, despite adequate volume resuscitation, hypotension persists, requiring vasopressors to maintain a mean arterial pressure ≥ 65 mmHg as was the case of our patient [2].

Clinically, sepsis presents with fever, tachycardia, and tachypnea as well as signs and symptoms relating to the organ bearing the source of infection [6]. Severe sepsis and septic shock cause decreased tissue perfusion leading to lactic acidosis, oliguria, and acute alteration in mentation [6,7]. The most commonly used scoring system to assess sepsis severity is the Sequential Organ Failure Assessment (SOFA) but the simplified qSOFA (quick SOFA) score can be used at the bedside to quickly assess and determine if a patient presenting with an infection is at risk of developing severe sepsis. Our patient had a score of 2, indicating a high risk for long ICU stays and high mortality [8]. 

Laboratory investigations for sepsis include a complete blood count, complete metabolic panel, lactic acid level, procalcitonin level, and C-reactive proteins [6]. Many different imaging modalities are also used to help localize the source of infection [3]. However, the culture of blood and body fluids is the gold standard for the identification of the microorganisms involved in sepsis, to inform the choice of antimicrobial [9]. Sometimes, evidence of infection is extrapolated from radiographic and laboratory results because as much as 30% of suspected sepsis may yield negative cultures [10]. Our patient had a negative blood culture, but his abdominal ultrasound was significant for cholecystitis.

Sepsis is a medical emergency and therefore requires timely initiation of aggressive and appropriate treatment [3,9]. Infection control, hemodynamic stabilization, and modulation of the septic response form the basis of treatment. Hypotension and perfusion defects are managed with fluids and pressors as needed [11]. Empirical antibiotic therapy should be started within 60 minutes of suspicion of sepsis, based on likely pathogen and local resistance patterns, and then de-escalated after identification of a causative organism [9,11]. Respiratory support becomes necessary and patients might undergo endotracheal intubation in cases of severe hypoxemia or severe depression of mentation [7].

Severe sepsis and septic shock as in the case of our patient can cause multiorgan failure with a very high risk of mortality [9]. In addition to local organ damage by the pathogens and the consequences resulting from organ hypoperfusion, sepsis has organ-specific mechanisms of causing damage to individual organs [7]. Our patient had multisystemic failure including the hematological system [12], cardiovascular system [13], central nervous system [7], hepatobiliary system [14], renal system [15], respiratory system [7], and musculoskeletal system [16].

It is worth noting that our patient’s laboratory findings were also significant for hyperammonemia. From the literature, about 90% of hyperammonemia is caused by liver dysfunction. Nonhepatic causes of hyperammonemia include inborn errors of metabolism, excessive muscle contractions, medications, malignancy, or infection with urease-producing bacteria [17]. Our patient was later diagnosed with late-onset OTCD. This is the most common urea cycle enzyme disorder worldwide and it occurs in about 1 in 56 500 people [4]. It is an x-linked disorder associated with markedly elevated ammonia levels. It has been observed that 30% of patients with OTCD present in the neonatal period while up to 70% present later in life [5]. Late-onset OTCD develops in men with partial OTCD and heterozygous women [5]. The median age of onset of late-onset OTCD is about 37 years [18]. 

Late-onset OTCD is difficult to diagnose because most people are asymptomatic or when symptoms develop, they are nonspecific with normal laboratory findings, except for hyperammonemia during crisis [4,5]. Symptoms usually develop after a stressful event like an acute infection, trauma, steroid administration, starvation, surgery, medications, or increased protein intake [4,18]. Patients may report chronic headaches, anorexia, and vomiting. Severe manifestations include confusion, seizures, and coma [18-20]. Ammonia crosses the blood-brain barrier and is metabolized by glutamine synthetase (GS). However, when large amounts of ammonia accumulate, it causes osmotic astrocyte swelling and cerebral edema due to intracellular accumulation of glutamine [21].

Our patient’s family reported that he was generally in good health before the present hospitalization. Although our patient had hepatitis B, he had normal liver enzymes upon admission, and imaging was unremarkable for any liver dysfunction. We can therefore conclude that his high ammonia level was likely due to an extrahepatic cause, in this case, OTCD, with sepsis being the precipitating factor possibly through the following mechanisms. Sepsis can cause liver dysfunction which can lead to abnormalities in ammonia metabolism [22]. Sepsis may also disrupt the intestinal barrier function which can lead to additional absorption of ammonia. Additionally, patients with sepsis are in a catabolic state, breaking down massive amounts of amino acids, leading to an increase in the production of ammonia [23]. Contributory factors to high ammonia levels in our patient were constipation [20] and tonic-clonic seizures [24]. In a patient deficient in an enzyme participating in breaking down ammonia in the urea cycle like the case of our patient who had OTCD, ammonia levels rapidly increase leading to hyperammonemic crisis [25]. Therefore, OTCD should be suspected in patients with marked hyperammonemia without liver cirrhosis. Of note, laboratory diagnosis of OTCD includes a low plasma citrulline level with increased urine orotic acid and it is confirmed with molecular genetic testing [26].

Ammonia levels must be rapidly decreased to levels below 200 µmol/L, as higher levels are associated with lower survival rates [4]. Patients with marked hyperammonemia should immediately undergo hemodialysis regardless of the cause [27]. Treatment also includes lactulose and rifaximin, ammonia scavengers like sodium phenylacetate, glycerol phenylbutyrate, and sodium benzoate, and supplementation with urea cycle intermediates like L-arginine and L-carnitine as well as low protein diet [28].

Our patient benefited from endotracheal intubation, HD, lactulose, antibiotics, fluid resuscitation, vasopressors, and transfusion of blood products. However, because of the many poor prognostic features present, severe acute kidney injury [29], severe hyperammonemia with change in mental status [4], severe rhabdomyolysis, and septic shock [16], the patient’s chances of clinical improvement were low.

## Conclusions

Sepsis is a major cause of morbidity and mortality and when suspected needs to be treated aggressively. With delayed treatment, multiorgan failure can develop. Additionally, sepsis can unmask diseases, like in the case of our patient, in which sepsis precipitated a hyperammonemia crisis and unmasked a rare adult onset OTCD. Consequently, clinicians should always consider OTCD as a differential diagnosis in patients presenting with severe hyperammonemia with normal liver function.
